# Mapping residual stresses in AlSi10Mg alloy fabricated by powder bed fusion-laser beam method

**DOI:** 10.1038/s41598-025-09316-0

**Published:** 2025-07-09

**Authors:** Mohamed E. Daoud, Inas Taha, Ahmed Abdelgawad, Imad Barsoum, Kamran A. Khan, Dalaver H. Anjum

**Affiliations:** 1 Department of Physics, Khalifa University, P. O. Box 127788, Abu Dhabi, United Arab Emirates; 2 Department of Mechanical Engineering, Khalifa University, P. O. Box 12788, Abu Dhabi, United Arab Emirates; 3 Aerospace Engineering Department, Khalifa University, P. O. Box 127788, Abu Dhabi, United Arab Emirates

**Keywords:** Residual stresses, Powder bed fusion-laser beam, AlSi10Mg alloy, Anisotropy, Transmission Electron microscopy, Finite element method, Characterization and analytical techniques, Transmission electron microscopy, Metals and alloys, Mechanical properties

## Abstract

Residual stresses in metal alloys fabricated by the powder bed fusion-laser beam (PBF-LB) method exhibit anisotropy influenced by laser scanning and building orientations. This study maps nanoscale residual stresses within a single grain of PBF-LB AlSi10Mg alloy using various transmission electron microscopy (TEM) modalities. Residual stress maps were obtained for both as-built and T6 heat-treated samples along scanning and building orientations. Post-analysis revealed that T6 heat treatment reduced tensile stress compared to as-built samples. For example, in the [1̅00] crystallographic direction, average tensile and compressive residual stresses in the building direction decreased by ~ 70% after T6 treatment. Anisotropy in residual stresses was also observed; in the [1̅00] direction, average residual stresses in scanning orientation were ~ 13% and ~ 23% higher than the building direction in as-built and T6-treated samples, respectively. Heat treatment effects were further examined using image-based finite element method (FEM) simulations to understand the stress-driven mechanisms behind Si eutectic break-up and Si precipitate diffusion into the Al matrix. The results revealed regions of tensile and compressive stress within Si eutectic zones, identifying them as sources for Si diffusion and nucleation of Si-based precipitates within the matrix.

## Introduction

Over the past two decades, Additive Manufacturing (AM) has become prominent among various manufacturing techniques, such as casting, molding, forging, and machining. This innovative method constructs three-dimensional (3D) objects by systematically layering material based on digital 3D computer-aided design (CAD) models. Unlike traditional methods that remove material, they rely on energy-intensive processes. AM offers a more efficient approach to handling raw materials and crafting complex components^1–3^. Among the diverse techniques within the AM domain, the powder bed fusion-laser beam (PBF-LB), is renowned for its capability to produce near-net-shape metal components with intricate geometries^3–8^. In PBF-LB, a high-energy laser meticulously traces and melts metal powder, layer by layer, based on CAD data, culminating in a three-dimensional structure^9–11^. Metals commonly employed in this process include aluminum alloys^7,12^, titanium alloys^13,14^, stainless steel^15^, and high entropy alloys^16^. The microstructure and mechanical properties of the resultant components are influenced by various parameters, such as laser power, scanning speed, layer thickness, hatch spacing, and the metal powder’s inherent characteristics^3,17,18^. Optimizing these parameters can significantly enhance the final product’s quality. The AlSi10Mg alloy, with its high specific strength, corrosion resistance, and exceptional thermal properties, has garnered significant interest for applications in the automotive and aerospace sectors. While many commercial aluminum alloys are not ideally suited for additive manufacturing (AM), AlSi10Mg has emerged as a favorable option due to its high powder flowability, narrow solidification range, and low shrinkage. Additionally, the eutectic composition of aluminum and silicon provides excellent weldability, making AlSi10Mg particularly attractive for industrial applications^19,20^.

Residual stresses (RS) refer to stresses that remain within a material even in the absence of external forces or applied loads. RS is typically classified according to its characteristic length scale: Type I stresses, or macro residual stresses, occur at the scale of the entire component and can cause significant distortions, particularly under changing boundary conditions. Type II stresses, often referred to as intergranular or micro stresses, occur at the grain level and are influenced by factors such as material anisotropy and variations in grain orientation. Type III stresses operate at the atomic scale and arise from atomic-level irregularities, such as vacancies or substitutional atoms^21–25^.

In additive manufacturing processes powder bed fusion-laser beam (PBF-LB), residual stresses mainly develop due to steep thermal gradients generated during rapid heating and cooling, resulting in localized thermal expansions constrained by the surrounding material^26^. Process parameters such as laser power, scanning speed, scanning strategy, layer thickness, and post-processing heat treatments strongly affect the residual stress states^27–32^. Various techniques, broadly classified as destructive (e.g., hole drilling, contour, curvature methods) and non-destructive (e.g., X-ray diffraction, neutron diffraction, ultrasonic and magnetic methods), have been developed to measure residual stresses^24,25,33–36^. However, most of these techniques primarily measure Type I residual stresses, which have been extensively studied in the literature^24,25,33–36^. Type II and III residual stresses, originating from local microstructural features and intergranular and intragranular interactions, play a crucial role in phenomena such as fatigue crack initiation and short crack propagation, which are largely governed by grain-level processes^37^. Nevertheless, the current literature’s investigation of Type II and III residual stresses remains limited.

Recent advances have significantly improved the understanding and prediction of residual stresses in metal additive manufacturing. Early investigations revealed the critical role of thermal gradients in stress formation^21,22^. Finite element methods (FEM) have been increasingly employed to model these phenomena with approaches accounting for layer-wise deposition, thermal cycling, and mechanical relaxation^23^. Experimental validation of FEM models, using methods such as industrial-Computed Tomography has been crucial to enhance model fidelity^24^. Recent developments have introduced image-based finite element modeling (FEM) techniques that utilize high-resolution microstructural data obtained through scanning electron microscopy (SEM)^25^. This approach enables a more precise representation of the material’s inherent heterogeneity, thereby enhancing the accuracy of residual stress distribution predictions and facilitating improved control over the mechanical performance of AlSi10Mg components fabricated via powder bed fusion–laser beam (PBF-LB) processes. However, to date, no studies have been reported on the application of image-based FEM using transmission electron microscopy (TEM) data le to predict the stress fields responsible for the experimentally observed microstructural features at the nanoscale. This gap in the literature forms the primary motivation for the present study.

This work employs transmission electron microscopy (TEM)-based techniques to map Type II residual stresses at the nanoscale. Specifically, a combination of four-dimensional scanning transmission electron microscopy (4DSTEM) and STEM-EELS spectrum imaging is used to characterize the residual stress distributions within individual grains of as-built and T6 heat-treated AlSi10Mg samples produced by PBF-LB. This methodology enables high-resolution, local analysis of residual stress states, providing new insights into the behavior of Type II residual stresses in additively manufactured Al alloys.

The experimental observations obtained by electron microscopy based modalities have been validated using image based finite element method (FEM) simulations which has been have been extensively employed to predict these residual stresses, enabling optimization of process parameters to mitigate adverse effects^26,27^. The image-based finite element method (FEM) was applied using the ABAQUS package to simulate residual stress maps for a deeper understanding of the mechanisms behind observations made by using the electron microscopy technique. The mechanisms that control residual stress distributions in PBF-LB synthesized AlSi10Mg alloys include the dissolution of Si eutectic, Si spheroidization, and creation of intermetallic precipitates in AlSi10Mg alloys after the heat treatment^28,29^.

## Materials and methods

### Sample preparation

The alloy samples were fabricated using an EOS M400-4 metal 3D printer (EOS GmbH, Germany) with a 400 W laser. The AlSi10Mg powder was provided by Powder Alloy Corporation (USA). The powder alloy’s elemental composition (wt%) was Si 9.0–11. 0, Mg 0.20–0.45, Fe ≤ 0.55, Mn ≤ 0.45, Cu ≤ 0.05, Ti ≤ 0.15, Ni ≤ 0.05, Pb ≤ 0.05, Zn ≤ 0.10, Sn ≤ 0.05, and Al the balance. Before synthesizing the samples, the as-received powder samples were characterized using a scanning electron microscope (SEM) and X-ray diffraction (XRD) to determine the size and crystal structures of powder particles, respectively. It is to be noted that the SEM analysis was performed by operating the microscopes at an accelerating voltage of 30 kV, and the images were generated by collecting the secondary electron signals. Whereas the XRD analysis of samples was performed at room temperature using a XRD PANalytical Empyrean with Cu Kα radiation of wavelength (λ = 1.54056Å) operating at 45 keV and 40 mA, covering a 2θ angle range from 10° to 90° with an increment of 0.01°.

**Fig. 1:**
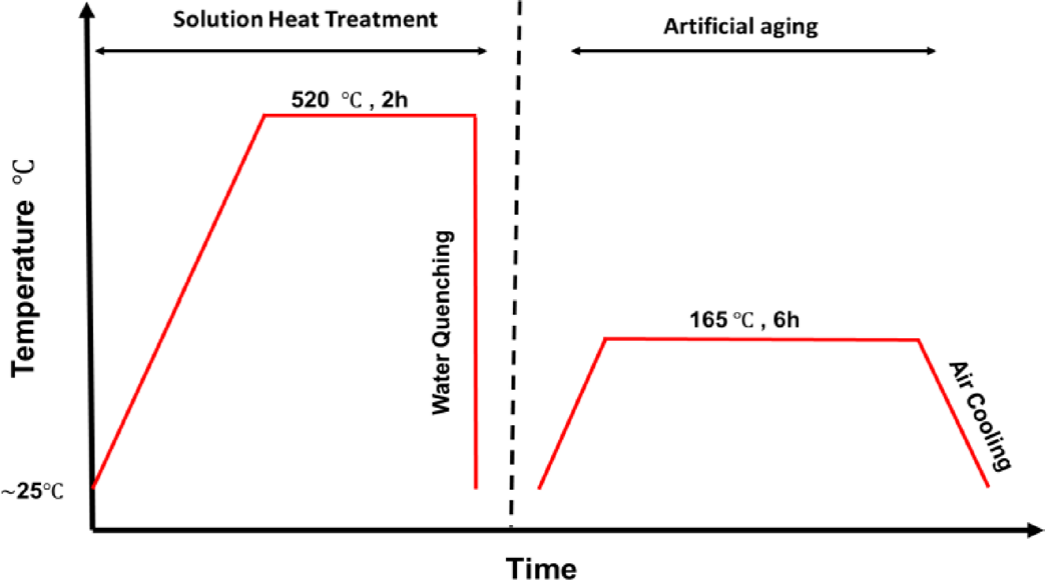
T6 heat treatment procedure for annealing of AlSi10Mg alloy samples.

The process parameters used during the PBF-LB fabrication of alloy samples were 400 W laser power, a scan speed of 1300 mm/s, 19 μm hatch spacing, 30 μm layer thickness, and a bi-directional scan strategy. The As-built samples were heat treated by following a T6 heat procedure, which consists of a solution heat treatment (SHT) for two hrs. at a temperature of 520 ºC followed by water quenching at room temperature ($\:\sim25$ºC) and finally by artificial aging (AA) for six hrs. at 165 ºC. Figure 1 illustrates the applied T6 heat treatment to demonstrate the procedure clearly. The storage time between quenching the samples and starting the aging process was minimal (~1 h).

### TEM lamellae preparation

**Fig. 2:**
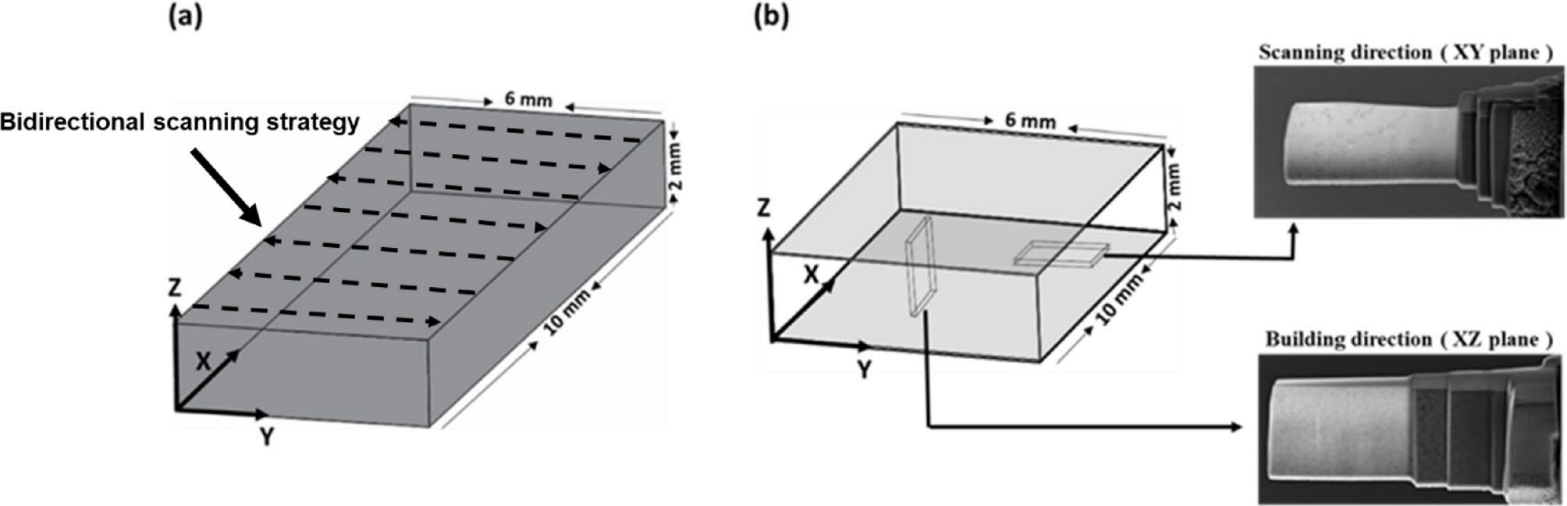
The process of preparing TEM lamellas from the AlSi10Mg sample. (**a**) The dimension of the AlSi10Mg sample, (**b**) TEM lamellas from the XZ plane (or building direction) and the XY plane (or scanning direction).

For the nanoscale mapping of residual stresses in samples with TEM, a couple of lamellae were prepared for each sample, i.e., in the alloy building orientation (Z-direction or XY plane) and in the laser scanning orientation (Y-direction or XZ plane). The details on the building, scanning orientation, and their subsequent lamellae preparation are schematically presented in Fig. 2. Several rectangular beam samples with dimensions of 100 × 6 × 2.5 mm^3^ were produced (see Fig. 2 (a)). Next, the pieces with 10 × 6 × 2 mm^3^ dimensions were cut from the beam samples for their characterization. The TEM lamellae were prepared using a Helios Nano Lab 650 FIB/SEM dual-beam system from ThermoFisher Scientific. To protect the top surface of the sample, platinum (Pt) layers were deposited on the surface region of interest using electron and ion beam techniques. The samples were then gradually thinned to a relative thickness of 100 nm by progressively reducing the ion beam energies in the FIB until reaching 2.0 keV. In this way, the gallium (Ga) ion implantation and the amorphization of the lamella surfaces were minimized.

### TEM data acquisition

**Fig. 3:**
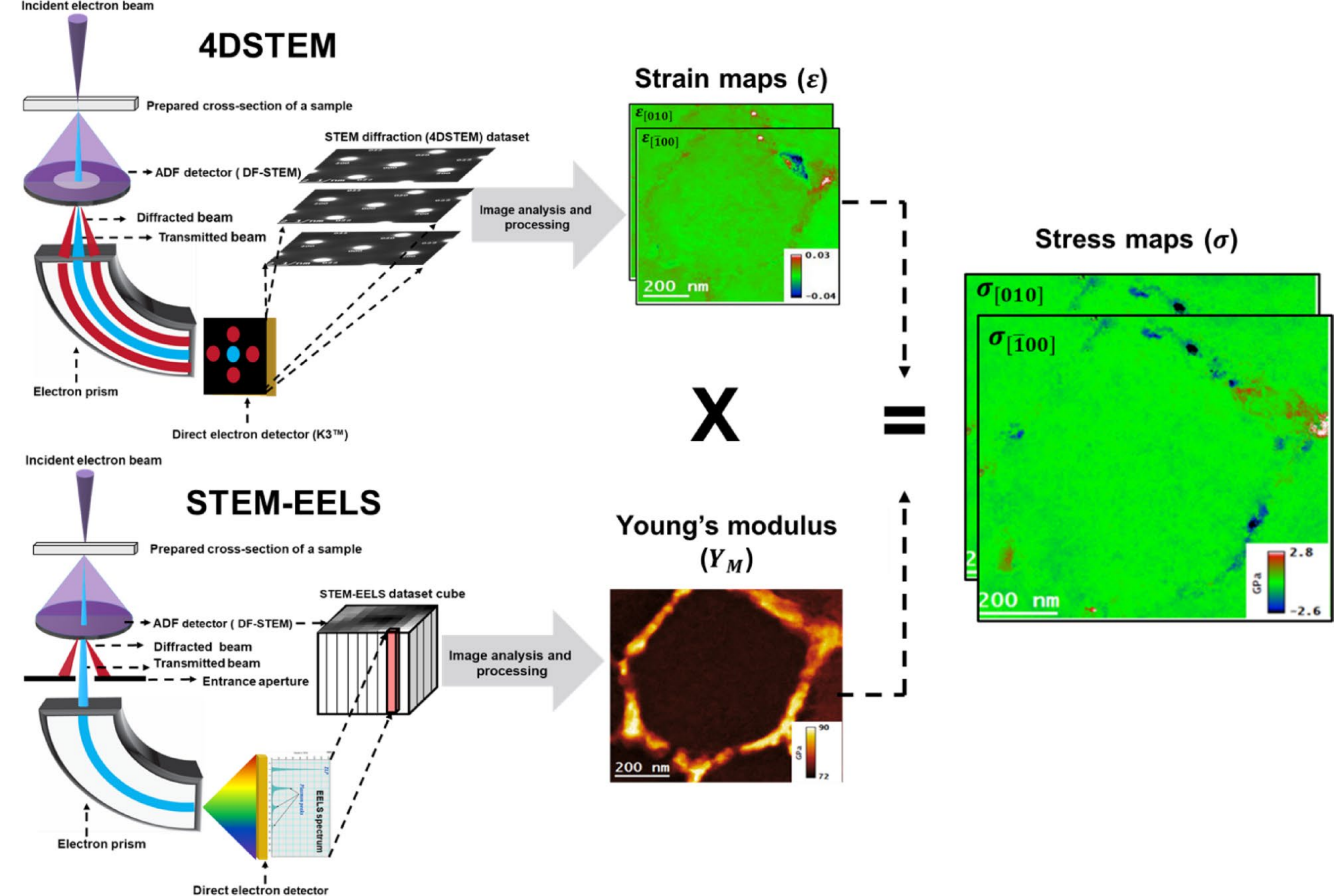
The methodology utilized to generate residual stress maps based on TEM techniques. (**a**) 4DSTEM techniques and the produced stain maps, (**b**) STEM-EELS technique and Young’s modulus map, and (**c**) the generated stress maps by multiplying the strain and Young’s modulus maps.

The prepared specimens were analyzed using a double aberration-corrected TEM of model Titan ThemisZ, also from ThermoFisher Scientific, which was equipped with an energy filter of model GIF Continuum 1069 h with K3 detector. The accelerating voltage of 300 kV was set during the mentioned TEM analysis. The microscope was aligned in STEM mode to have a spherical aberration coefficient (Cs) of ~ 2 μm to reach the point resolution of about 70 pm. Moreover, a high-angle annular dark-field (HAADF) detector produced images of conventional dark-field scanning transmission electron microscopy (DF-STEM). The microscope was set to microprobe STEM mode at the convergence angle of 0.70 mrad while acquiring 4DSTEM datasets. The diffraction patterns were collected using a K3 direct electron detector in image mode (with binning 4, size 1/4) to gain high speed and low signal-to-noise ratio in the patterns. During STEM-diffraction (4DSTEM) data acquisition, an energy slit of width 20 eV was inserted in the back focal plane of the electron prism to remove diffuse scattering contributions in the acquired diffraction pattern. The 4DSTEM datasets were acquired by tilting the specimen in the [001] direction. The Young’s modulus $\:{(Y}_{M})$ maps were generated by acquiring the low-loss EELS datasets in microprobe DF-STEM mode by tilting the specimen in the same [001] direction. The pixel size in acquired STEM-EELS datasets was around 2 nm, while EELS datasets were acquired with a dispersion of 30 meV eV per channel. Furthermore, the collection angle (β) for the acquisition of EELS spectra was ~ 20 mrad by setting the camera length of 37 mm and GIF entrance aperture of 5 mm size. Additionally, the aperture size of 70 μm for the second condenser lens (C2) was used during the data acquisition. In addition, X-ray energy dispersive (EDS) datasets were collected using the high-efficiency 4-sector EDS detector known as the SuperX, provided by ThermoFisher Scientific, having a total solid angle of 0.8 steradians. Figure 3, presents the methodology employed to generate residual stress maps.

### Post-processing of TEM data

Four-dimensional scanning transmission electron microscopy (4DSTEM) involves acquiring a two-dimensional diffraction pattern at each pixel of a dark-field scanning TEM (DF-STEM) image^21^. As the electron beam scans pixel-by-pixel, residual strains cause shifts in the diffraction spots or discs. By measuring these shifts, the perpendicular components of the displacement vector, u_x_ and u_y_​, can be determined. The derivatives of these displacement components yield the corresponding strain components, calculated using the following relations^22^:

1 $$\:{\epsilon\:}_{xx}=\:\frac{\partial\:{u}_{x}}{\partial\:x},\:\:{\epsilon\:}_{yy}=\:\frac{\partial\:{u}_{y}}{\partial\:y},{\epsilon\:}_{xy}=\frac{1}{2}\:\left[\frac{\partial\:{u}_{x}}{\partial\:x}+\frac{\partial\:{u}_{y}}{\partial\:y}\right]$$

where $\:{\epsilon\:}_{xx}$, $\:{\epsilon\:}_{yy}$ are the normal strain components and $\:{\epsilon\:}_{xy}$ is the shear strain component. Post-processing of the 4DSTEM datasets allows for the extraction of the $\:{u}_{x}$and $\:{u}_{y}$​ displacement fields are subsequently used to calculate the corresponding normal strain components. It is important to note that under the experimental conditions employed, the mapped normal strain component $\:{\epsilon\:}_{xx}$ and $\:{\epsilon\:}_{yy}$ correspond to the [1̅00] and [010] crystallographic directions, respectively. The acquired 4DSTEM datasets were post-processed to obtain Strain maps using the Gatan microscopy suite software (GMS 3.5 version) https://www.gatan.com/products/tem-analysis/gatan-microscopy-suite-software.

The residual stress maps can be determined directly by multiplying Eq. (3) with the literature value of the $\:{Y}_{M}$ under Hooke’s Law, which is given below:

2 $$\:{\sigma\:}_{ij}=\:{Y}_{M}\:{\epsilon\:}_{ij}$$

The validity of Eq. (2) holds as long as $\:{Y}_{M}$ is constant at each pixel of the DF-STEM image. The shear stress component is not addressed in the present study, as its analysis will be the focus of a separate, dedicated investigation. To determine more accurate residual stress maps, the determination of $\:{Y}_{M}$ must be done at the same pixel at which the residual strain is determined. The determination of the $\:{Y}_{M}$ can also be achieved by utilizing STEM-EELS Spectrum imaging(SI), which involves collecting an electron energy loss spectrum (EELS) at the corresponding pixel of the DF-STEM image. The acquisition of the EELS spectrum leads to the determination of plasmon energy at each image pixel, which can be utilized to determine the $\:{Y}_{M}$ locally. The following relation can be used to determine $\:{Y}_{M}$ from the plasmon energy $\:\left({E}_{p}\right)$ of the Al alloys. The value of *Y*_*M*_ at each pixel was determined from the measured *E*_*p*_ by using the following equation^30^:

3 $$\:\:\:{Y}_{M}=0.08\:{E}_{P}^{2.5}$$

The subsequent post-processing of the acquired STEM-EELS spectrum-imaging (SI) datasets was carried out using the non-linear least square (NLLS) method available in GMS 3.5 to obtain $\:{E}_{p}$ maps that contained information on the shift in $\:{E}_{p}$ values at each pixel of the dataset. The obtained $\:{E}_{p}$ maps were then further processed according to Eq. (3) to obtain the desired $\:{Y}_{M}$ maps. As stated above, the residual stress maps were then generated by multiplying the $\:{Y}_{M}$ maps and the corresponding residual strain maps. The residual stress maps can be determined directly from Eqs. (1) and (3) using Hooke’s Law, which is given by Eq. (2). The post-processing of STEM and STEM-EDS datasets was performed using the Velox Software Package version 3.15.0 (https://www.thermofisher.com/ae/en/home/electron-microscopy/products/software-em-3d-vis/velox-software.html).

### Methodology for FEM simulations

A commercial FEM software package named ABAQUS 2022 version (https://www.3ds.com/products/simulia/abaqus) was used to simulate the resulting residual stress in as-built samples due to T6 heat treatment. Temperature-dependent thermal and mechanical properties of Al and Si materials are listed in Table 1. It is to be noted that these properties were used in the FEM simulations. Additionally, the physical properties were normalized with their corresponding value at room temperature, and the temperature was normalized with the melting temperature of Al $\:\left({T}_{m}=660\:\right)$ºC and Si $\:({T}_{m}=1414\:)$ºC.

**Table 1:** Material properties at room temperature for the al matrix and the eutectic Si.

Property	Parameter	Al	Si
Melting temperature ($\:\text{K}$)	$\:{T}_{M}$	660	1414
Young’s Modulus (GPa)^31,32^	$\:{Y}_{{M}_{0}}$	69	130
Poisson ratio^31^^[,32^	*ν*	0.33	0.28
Specific heat (J K^−1^ kg^−1^)^31^^[,33^	$\:{C}_{{P}_{0}}$	900	715
Thermal conductivity (W K m^−1^)^31^^[,34^	*k*_*0*_	247	142.2
Thermal expansion (10^−6^ C^−1^)^31^^[,35^	*α*_*0*_	22.8	2.63

**Fig. 4:**
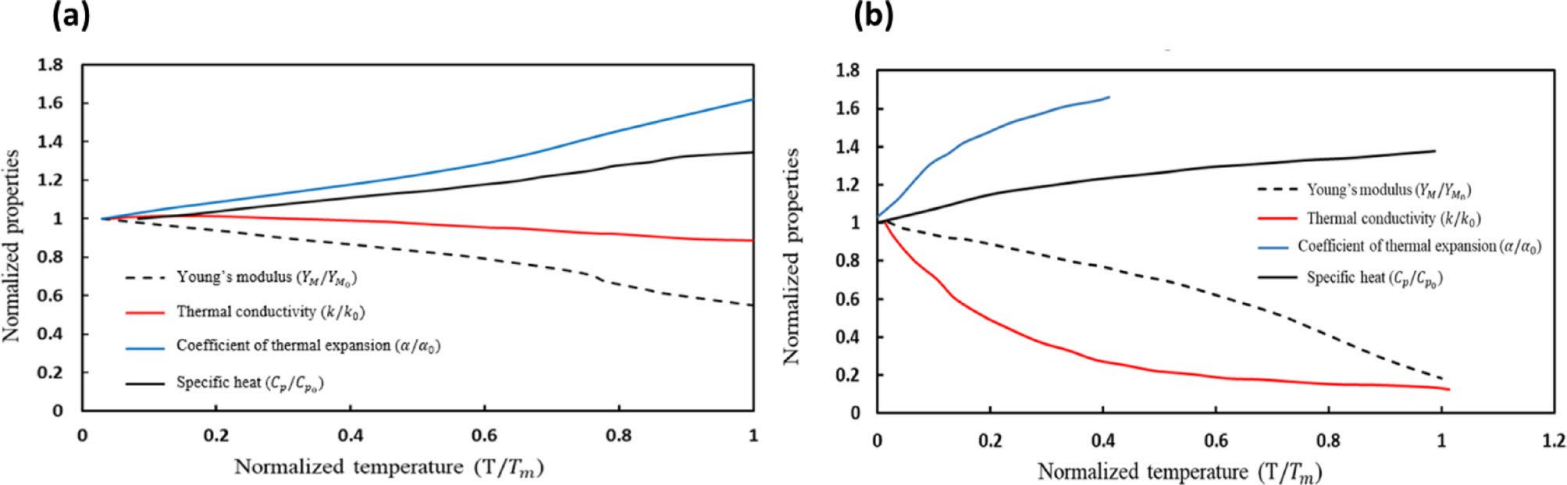
Temperature-dependent properties normalized with their room temperature values: (**a**) Al and (**b**) Si.

The normalized temperature-dependent properties of Al and Si were plotted by combining the data from the literature^31,34–41^. It was found that the dependence of materials properties as a function of temperature is non-linear for Al and Si (see Fig. 4).

**Fig. 5:**
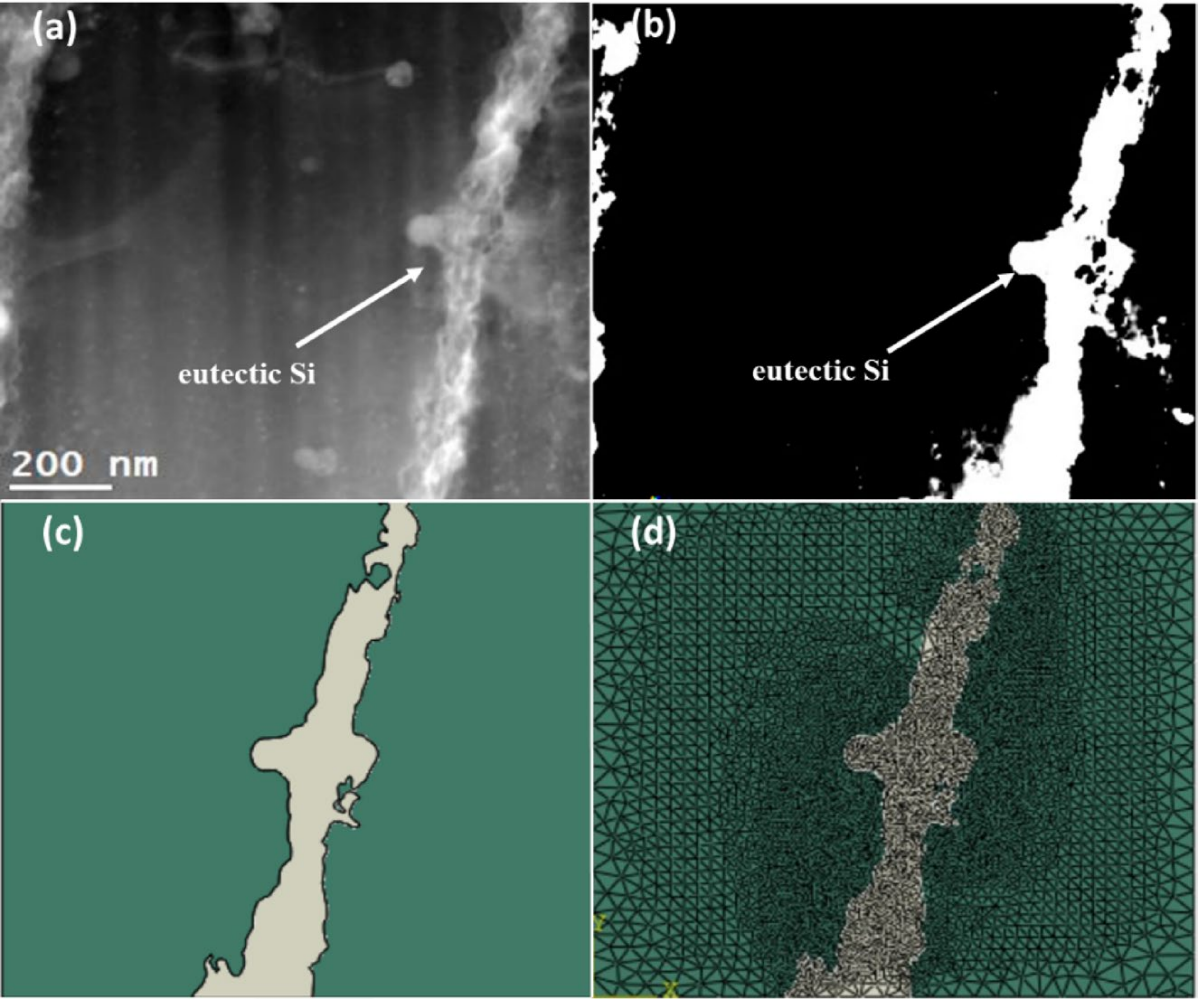
Image processing of the DF-STEM image of as-built AlSi10Mg alloy and the creation of the FE mesh. (**a**) DF-STEM image, (**b**) Processed image, (**c**) Generated 2D solid geometry in ABAQUS, and (**d**) Finite element mesh.

The T6 heat treatment was applied to the as-built sample in the laser scanning orientation during the simulations. Only the first stage of the T6, which is the solution heat treatment (from room temperature to $520$ºC) was considered, because at this stage the breaking of the Si eutectic and Si diffusion take place^42^. Also, we assume a < 100 > texture in the simulation. The steps followed in the utilized methodology for carrying out the simulations are presented in Fig. 5. Firstly, the DF-STEM image with 73000x magnification shows the eutectic Si boundary, and the Al matrix is presented in Fig. 5. The plane stress assumption, which postulates finite length in the third direction instead of infinite length in plane strain, contributes to a more precise representation. To ensure accuracy and stability in the results, elements with a minimum size of 5 nm were incorporated into the mesh, yielding an approximate total of 16,000 triangular elements for the model depicted in Fig. 5(d). A thermal cyclic loading was applied to all the elements to simulate the solution heat treatment step in T6 heat treatment. The heat treatment cycle consists of heating from 300 K to 793.15 K in 7200 s, then holding at 793.15 for 7200 s, and finally cooling to 300 K for 10s. The left boundary of the Representative Volume Element (RVE) is subject to constraints restricting movement along the X-direction or $\:\left[\stackrel{-}{1}00\right]$ direction while permitting movement along the Y-direction or$\:\:\left[010\right]$ direction. Conversely, the top boundary is constrained to movement along the Y-direction and permitted to move in the X-direction, ensuring uniform displacement for all nodes along the bottom boundary.

## Results and discussion

**Fig. 6:**
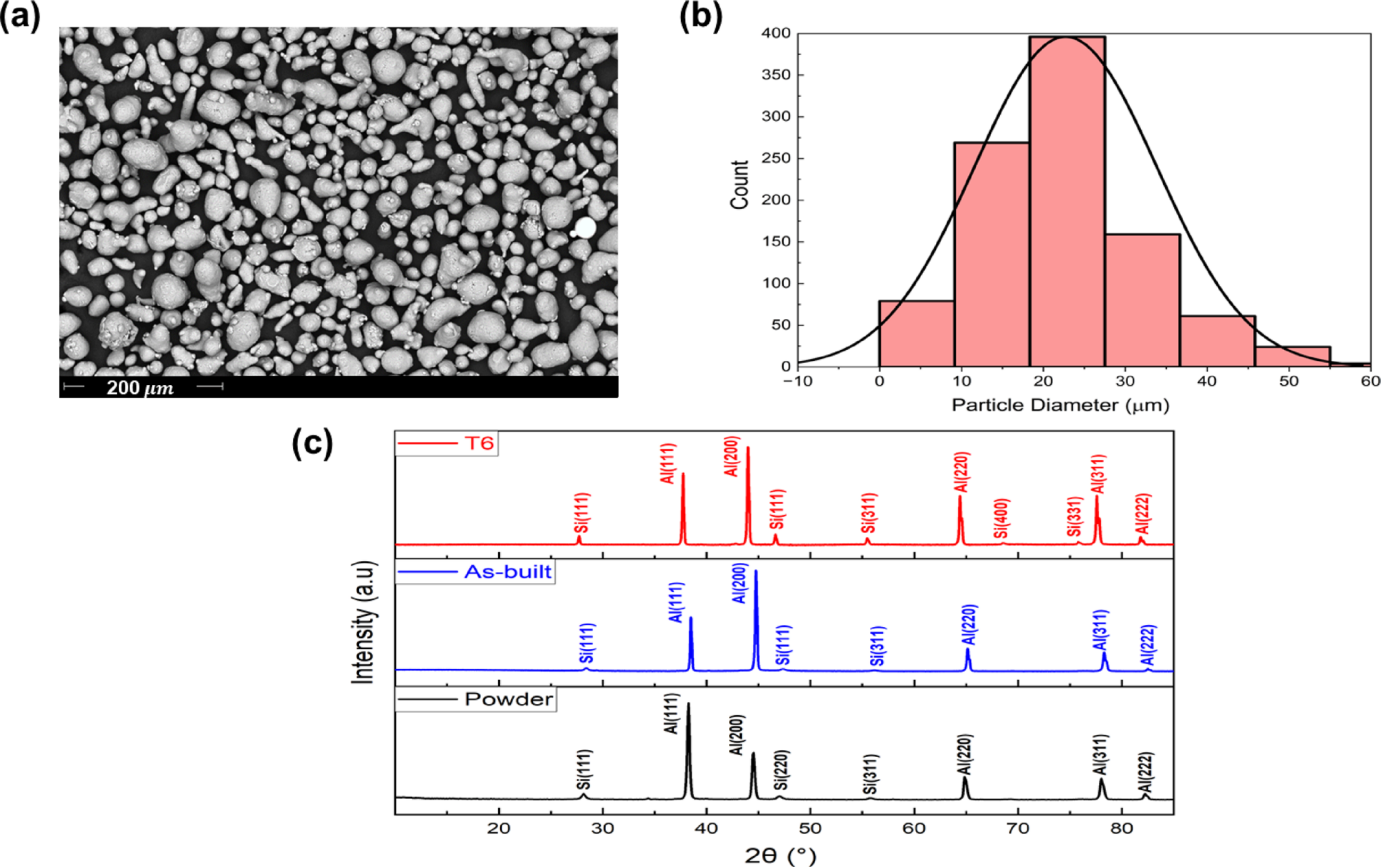
(**a**) SEM image of the morphology of AlSi10Mg powder used in the fabrication of the samples, (**b**) distribution of particle size, and (**c**) XRD peaks of AlSi10Mg powder, as-built, and T6 heat-treated samples.

The as-received AlSi10Mg powdered samples were analyzed using SEM and XRD techniques. The results are presented in Fig. 5. The samples had particles whose size was 5 to 60 μm, as confirmed from the SEM image shown in Fig. 6 (a). The particle size distribution analysis of SEM images was performed to find the average particle size, which was around 27 μm (Fig. 6 (b)). Similarly, XRD analysis of the as-received powder, as-built, and annealed samples was carried out, and the results obtained are presented in Fig. 6 (c). The XRD data showed that the crystal structure of the samples was face-centered cubic. Specifically, the acquired patterns predominantly showed diffraction peaks for the aluminum matrix and the eutectic Si phase. Still, peaks associated with the Mg_2_Si phase were not observed in as-built and T6 heat-treated samples. It can be attributed to a lower concentration of Mg relative to the Si element.

**Fig. 7:**
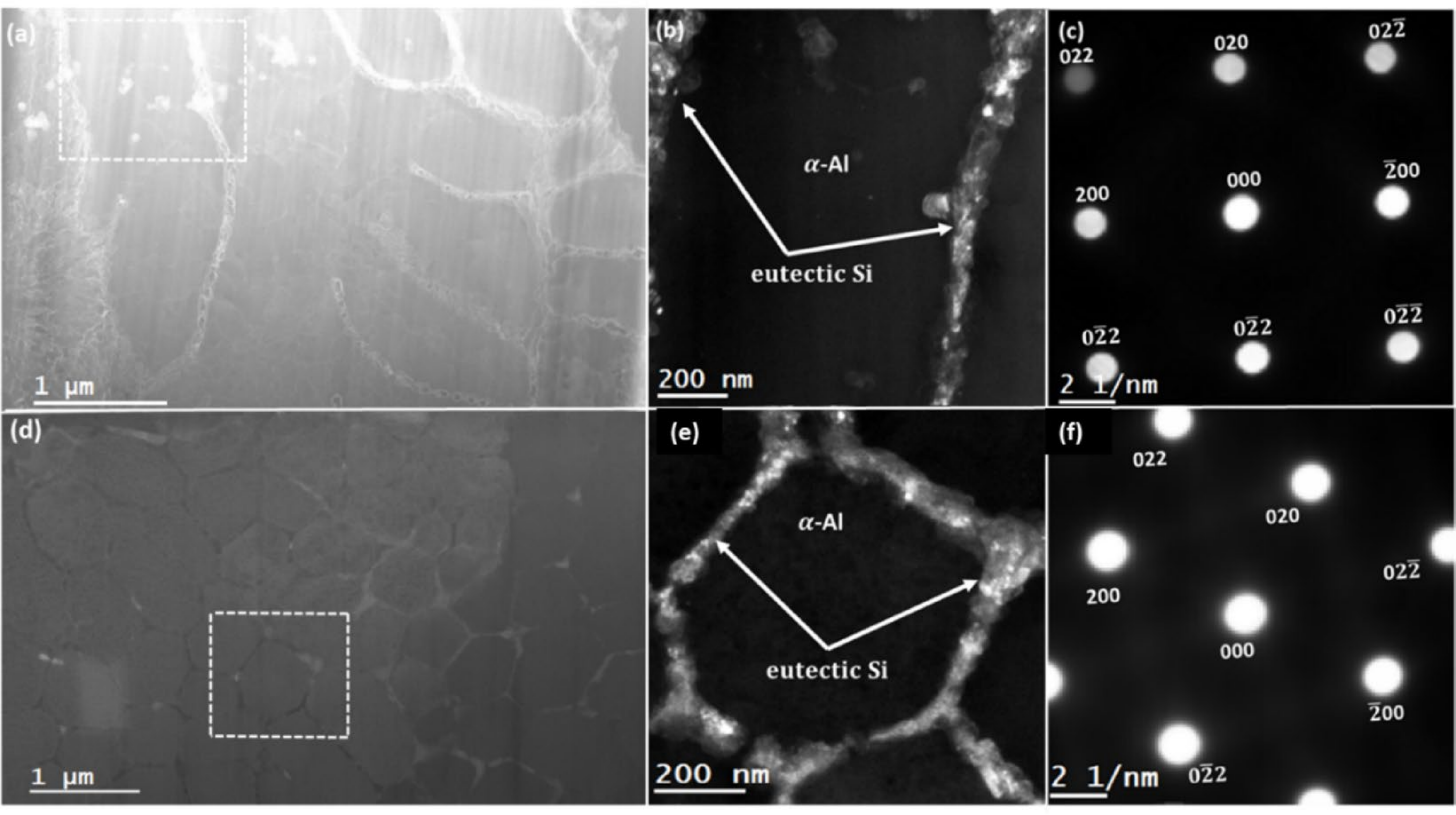
DF-STEM imaging analysis of as-built AlSi10Mg sample. (**a**) and (**d**): DF-STEM images showcasing the microstructure observed from specimens prepared in building and scanning orientations, respectively. (**b**) and (**e**): higher magnification DF-STEM images of specimens from building and scanning orientations. The presented images exhibit a contrast in the Si eutectic regions that possibly emanate due to a gradient in Si concentration. (**c**) and (**f**): Electron diffraction pattern of the specimen from building and scanning orientations, respectively. The acquired patterns confirm the tilting of the specimens along $\:{\left[001\right]}_{Al}$ zone axis.

**Fig. 8:**
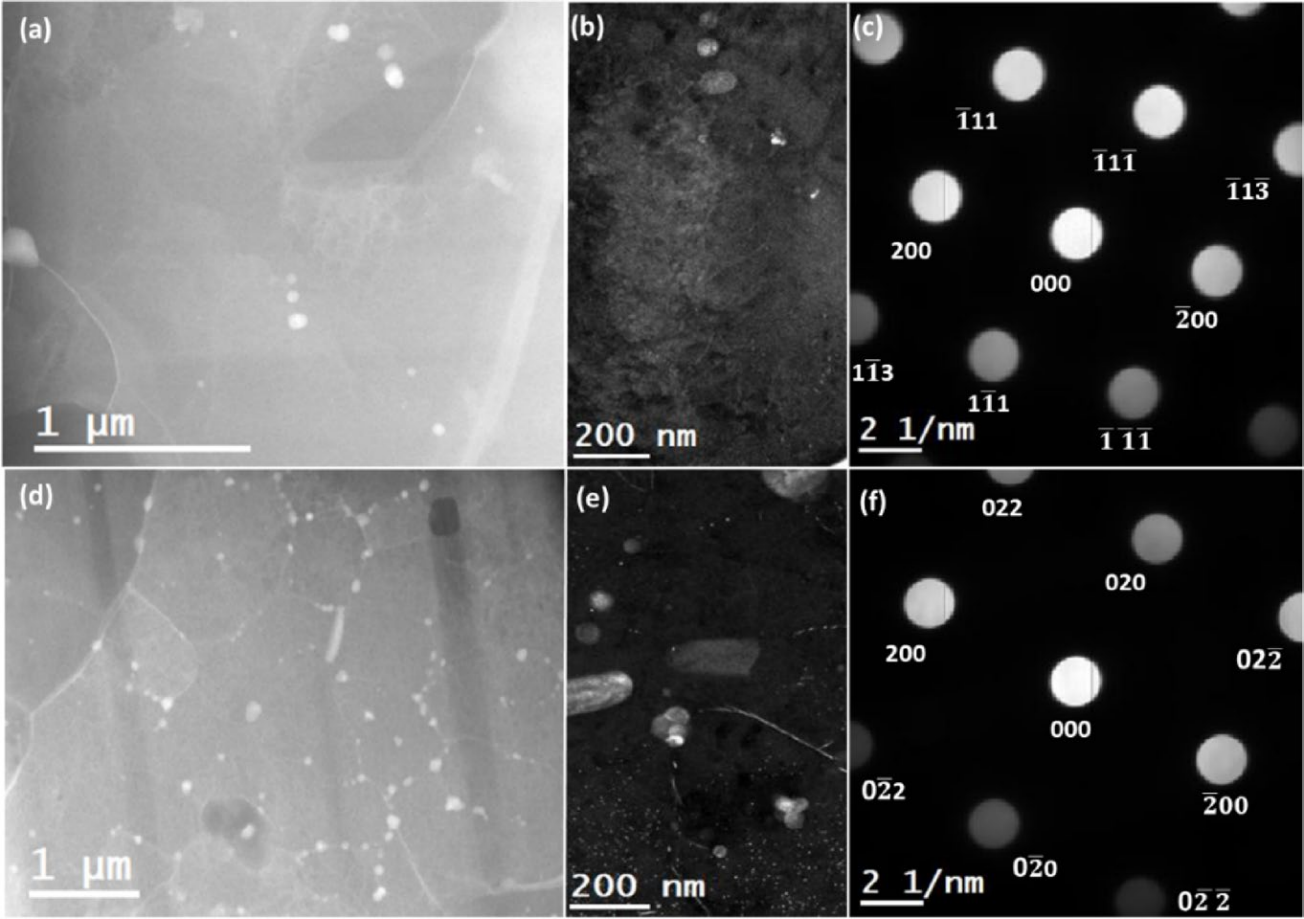
DF-STEM analysis of T6 heat-treated AlSi10Mg sample. (**a**) and (**d**): DF-STEM images showcasing the microstructure observed from specimens prepared in building and scanning orientations, respectively. (**b**) and (**e**): higher magnification DF-STEM images of specimens from building and scanning orientations. The presented images exhibit a contrast in the Si eutectic regions that possibly emanate due to a gradient in Si concentration. (**c**) and (**f**): Electron diffraction pattern of the specimen from building and scanning orientations, respectively. The acquired patterns confirm the tilting of the specimens along $\:{\left[011\right]}_{Al}$ zone axis and $\:{\left[001\right]}_{Al}$ zone axis for building and scanning orientations, respectively.

A detailed investigation of the alloy’s microstructure was conducted next by using the DF-STEM imaging technique, and the acquired results for as-built and T6 heat-treated samples are presented in Figs. 7 and 8, respectively. The DF-STEM images of the as-built samples in building and scanning orientations are presented in Fig. 7 (a) and (d), respectively. The images showcase the presence of the primary α-Al matrix and the Si eutectic network in the samples. Notably, the Si eutectic boundaries are more intense in the DF-STEM images than the Al matrix because electron scattering from Si is higher than from the Al matrix. The observed elongation agrees with the expected size of the Al grains that tend to stretch along the building direction, as it has been reported in the literature for the PBF-LB manufactured Al alloys^43^. To determine the crystal structure and strain in the samples, a specific grain was tilted along a certain zone axis, i.e., along $\:{\left[001\right]}_{Al}$ zone axis for as-built samples. The DF-STEM image in Fig. 7 (b) is acquired at higher magnification from a region enclosed by a dashed rectangular line. The corresponding diffraction pattern from the rectangular region is presented in Fig. 7 (c) and it confirms that it has been oriented along the $\:{\left[001\right]}_{Al}$ zone axis. Similarly, Fig. 6 (e) contains a higher magnification image of the rectangular region shown in Fig. 7 (b). The corresponding diffraction pattern from this rectangular region is presented in Fig. 7 (f), and it confirms $\:{\left[001\right]}_{Al}$ zone-axis orientation as well. In addition to confirming the zone-axis orientation of these regions, the presented DF-STEM in Fig. 7 (b) and (e) also shows the presence of sub-details in Si eutectic regions, alluding to the existence of a gradient in the Si concentration.

The DF-STEM analysis of the T6 heat-treated samples in scanning and building was carried out next, and the obtained results are presented in Fig. (8). The low-magnification DF-STEM images in Fig. 8 (a) and (d) show the effect of annealing on the microstructure of as-built alloy when the alloy is viewed from building and scanning orientations. The presented images highlight the dissolution of the eutectic Si boundaries and possibly the formation of Si precipitates in the Al matrix regions. The formation of the precipitates can be corroborated by the presence of white spherical-shaped particles by higher magnification DF-STEM images of the building and scanning orientation specimens, as shown in Fig. 8 (b) and (e), respectively. It is to be noted that these higher magnification DF-STEM images are acquired from the rectangular regions that are enclosed by dashed lines in Fig. 8 (a) and (d). Their corresponding electron diffraction patterns are presented in Fig. 8 (c) and (f). The electron diffraction patterns show the tilting of the scanning and building specimens along $\:{\left[011\right]}_{Al}$ zone axis and $\:{\left[001\right]}_{Al}$ zone axes, respectively.

**Fig. 9:**
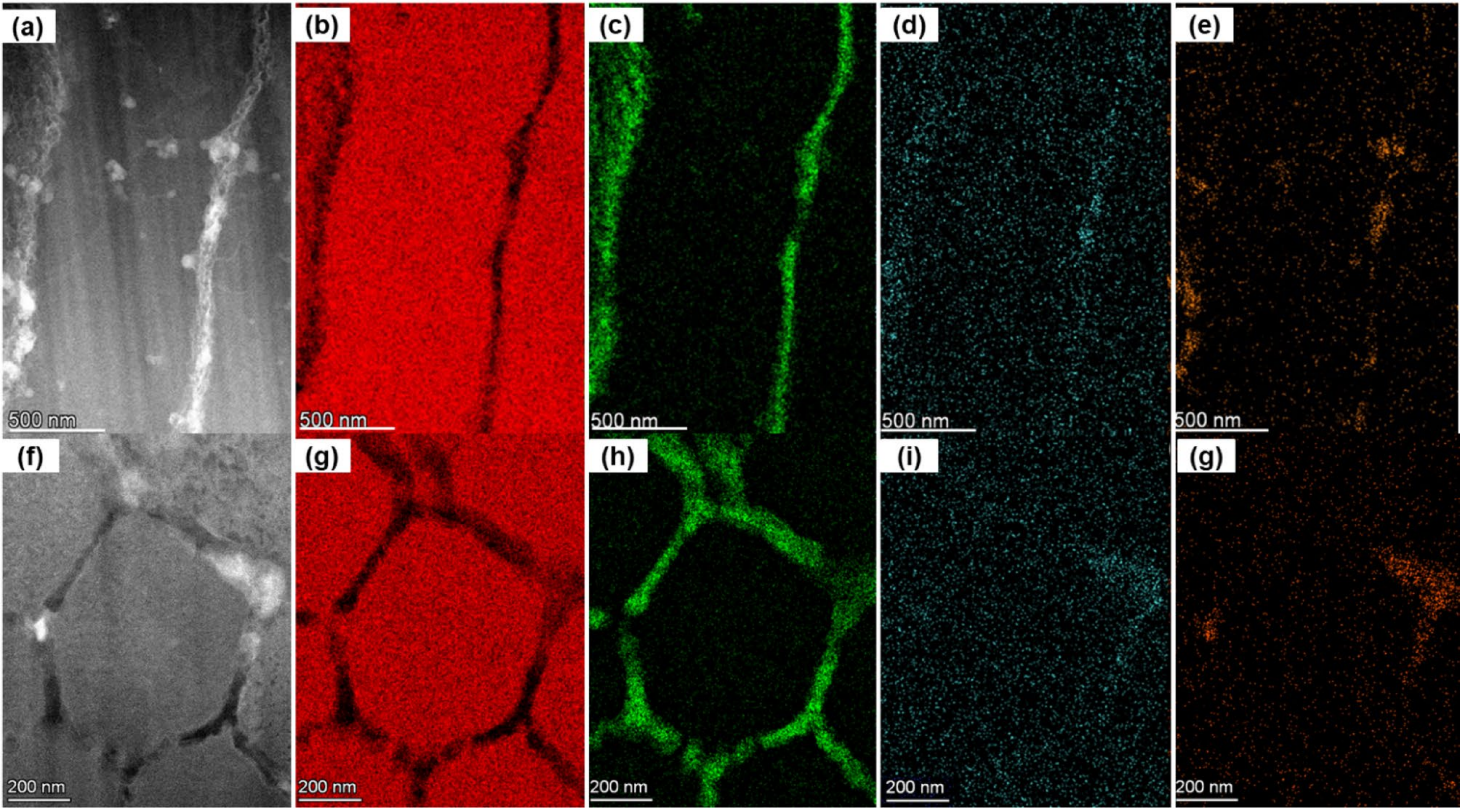
The STEM-EDS analysis for the determination of elemental distributions in as-built samples building and scanning orientation specimens. (**a**), and (**f**): DF-STEM images showcasing the regions selected for elemental analysis, (**b**), and (**g**): Al maps, (**c**), and (**h**): Si maps, (**d**), and (**i**): Mg maps, (**e**), and (**j**): Fe maps.

The elemental distributions or maps in the alloys play a significant role in giving rise to their residual stress distributions. Therefore, using the STEM-EDS technique, elemental distributions were acquired for both as-built and T6 heat-treated samples. The elemental mapping of both samples has been carried out from the same regions as shown in Fig. 7 (b), (e), Fig. 8 (b),and (e) so that a direct relationship of the microstructure of alloys can be established with their corresponding elemental distributions. It is to be noted that the elemental distribution of only Al, Si, Mg, and Fe elements was carried out by using the STEM-EDS technique, as the concentrations of the rest of the elements in the alloys were too low to be detected reliably. The results of the elemental distributions in as-built samples from scanning and building orientation specimens are presented in Fig. 9. Specifically, Figs. 9 (a-e) and (f-j) contain the results from specimens prepared in scanning and building orientation of the as-built samples. The presented results show that Si eutectic regions (see Fig. 9(a) and (f)) possess alloy elements, namely Si, Mg, and Fe, in higher concentrations than the Al matrix regions. Such higher concentration regions exist along the boundaries and profoundly affect the residual stress and the mechanical properties of the alloys^44^.

**Fig. 10:**
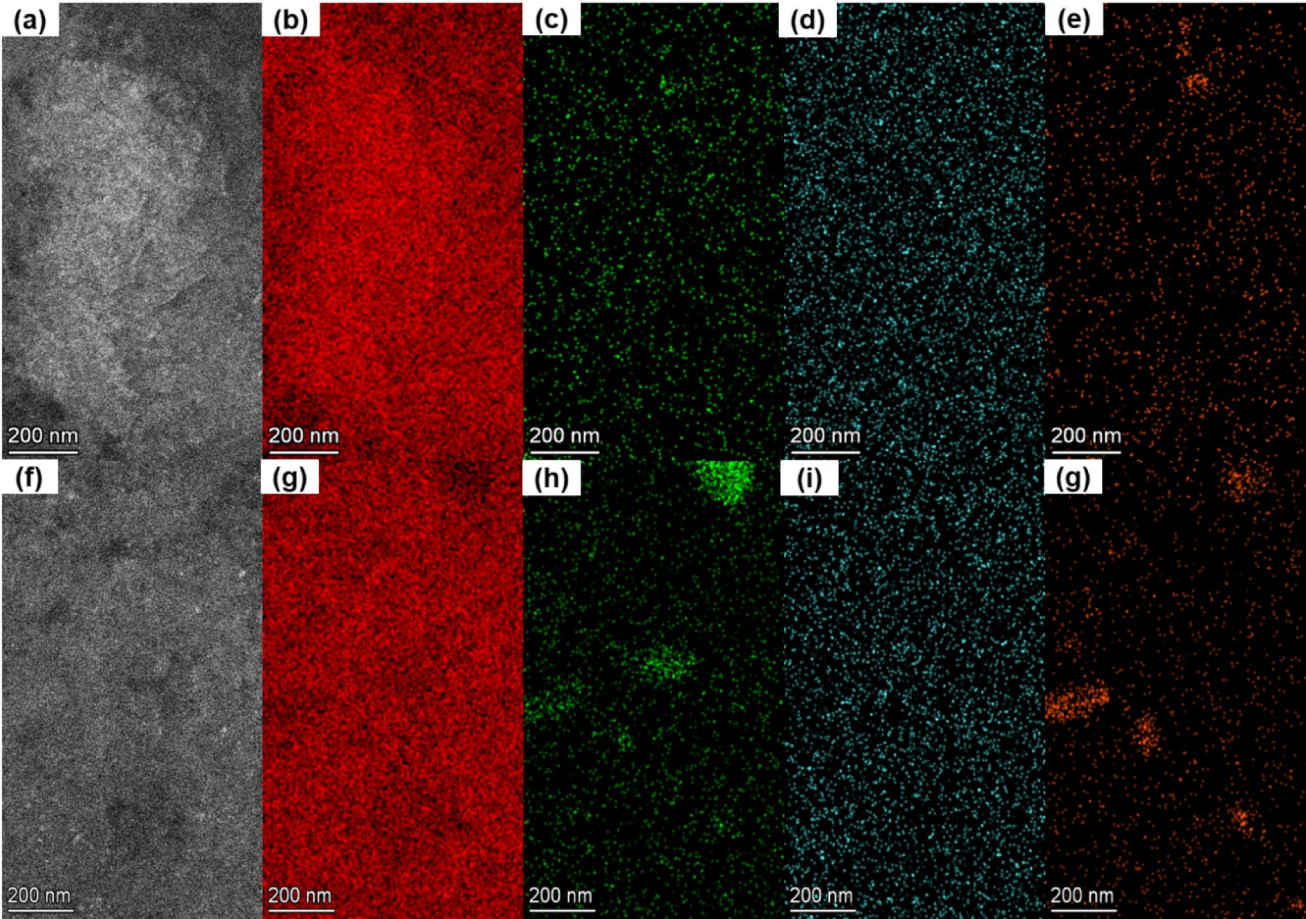
The STEM-EDS analysis for the determination of elemental distributions in T6 heat treated samples from building and scanning orientation specimens. (**a**), and (**f**): DF-STEM images showcasing the regions selected for elemental analysis, (**b**), and (**g**): Al maps, (**c**), and (**h**): Si maps, (**d**), and (**i**): Mg maps, (**e**), and (**j**): Fe maps.

The STEM-EDS analysis of T6 heat-treated AlSi10Mg alloy samples was carried out next, and the obtained results are presented in Fig. 10. The figure shows that the STEM-EDS elemental maps of the T6 heat-treated samples are from building and scanning orientation specimens, Fig. 10 (a-e), and (f-j), respectively. Segregated concentrations of alloy elements at the Si eutectic regions observed in the as-built samples were almost entirely dissolved as a result of annealing under the T6 treatment of the alloys. In other words, elemental distributions are presented in Fig. 10, indicating that the alloy elements diffused into the Al matrix regions. Ultimately, the diffusion of alloy elements into the matrix region will form precipitates due to the interplay among thermal-induced gradients in elemental compositions and residual stresses.

**Fig. 11:**
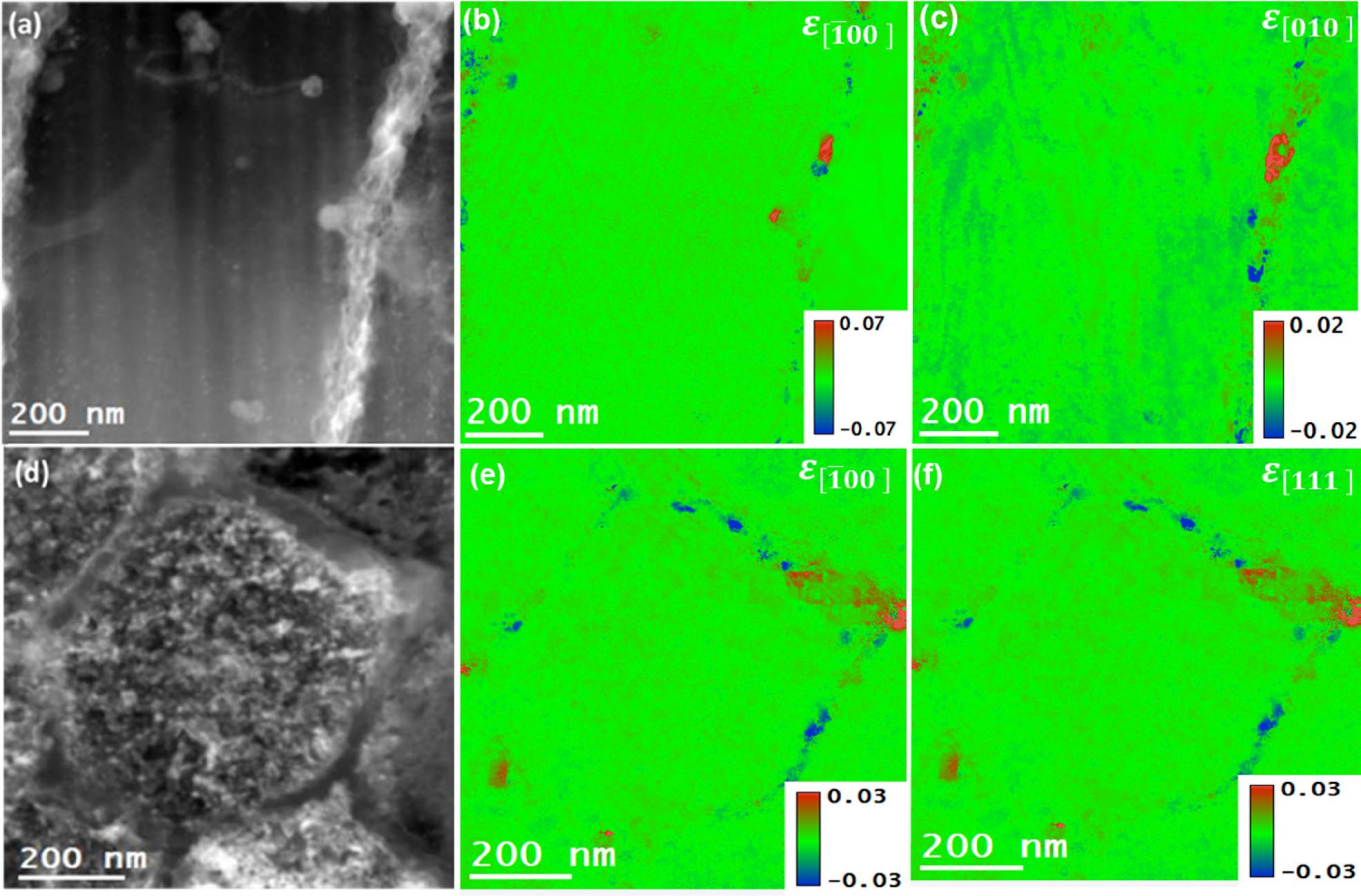
Strain analysis of as-built sample from specimens in building and scanning orientations by using 4DSTEM technique. (**a**), and (**d**): DF-STEM images showcasing the regions selected for strain analysis, (**b**), and (**e**): residual strain components (ε_xx_) in $\:\left[\stackrel{-}{1}00\right]\:$direction, (**c**), and (**f**): residual strain components (ε_yy_) in $\:\left[010\right]\:$direction.

**Fig. 12:**
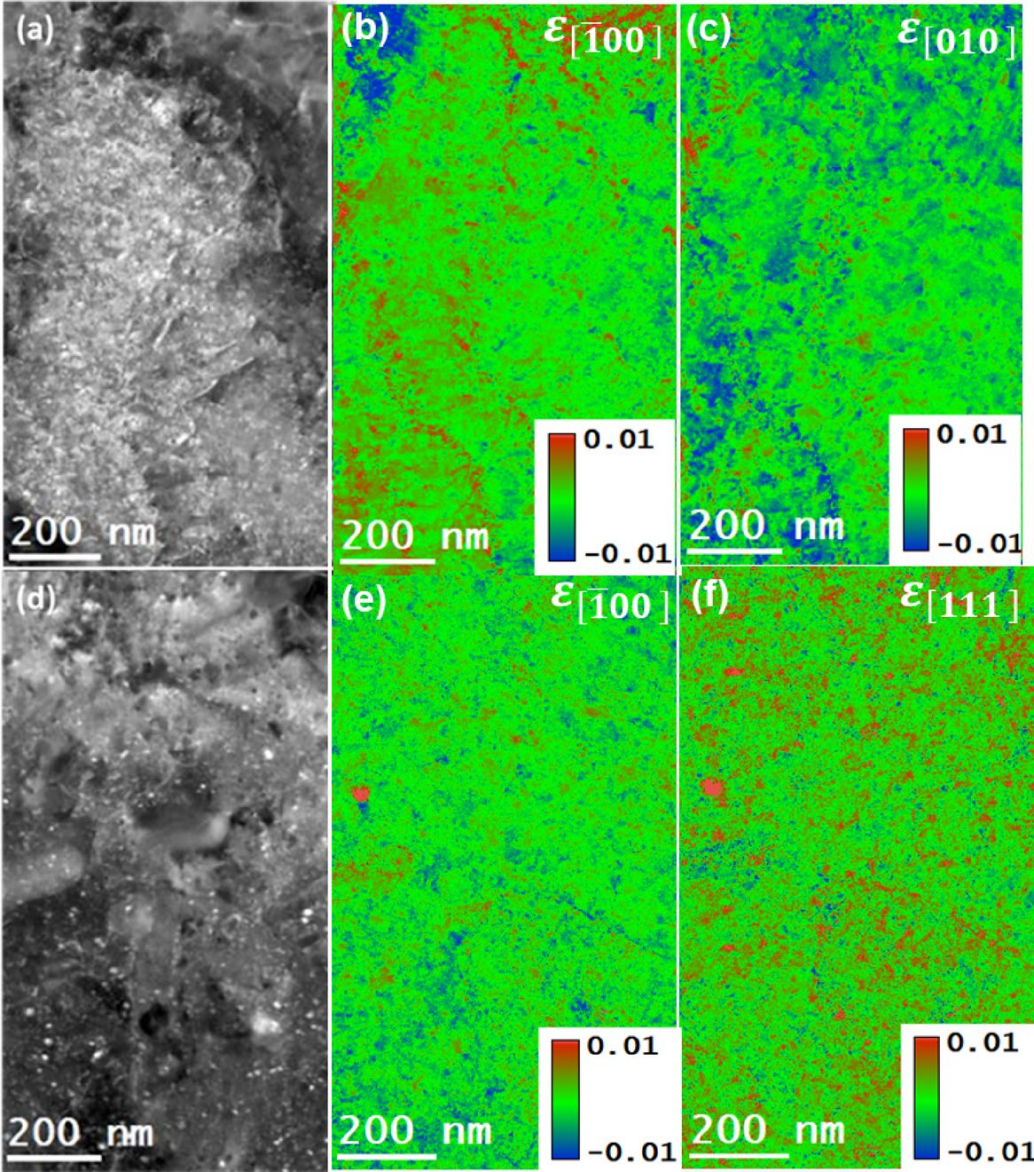
Strain analysis of T6 heat-treated sample from specimens in building and scanning orientations by using the 4DSTEM technique. (**a**), and (**d**): DF-STEM images showcasing the regions selected for strain analysis, (**b**), and (**e**): residual strain components (ε_xx_) in $\:\left[\stackrel{-}{1}00\right]\:$directions (**c**), and (**f**): residual strain components (ε_yy_) in $\:\left[1\stackrel{-}{1}1\right]$ and $\:\left[010\right]\:$directions.

The strain maps of AlSi10Mg alloy samples for as-built and after T6 heat treatment samples were meticulously generated by acquiring and post-processing the 4DSTEM datasets. The strain measurements were carried out from the same regions of the building and scanning orientation specimens from where their microstructure and elemental distributions were investigated. The residual strain results from as-built and T6 heat-treated samples are presented in Figs. 11 and 12, respectively. The acquired 4DSTEM datasets were post-processed in the STEMx package to compute residual strains numerically under the Eq. (4). This approach enabled the determination of normal strain components, ε_xx_ and ε_yy_, along specific crystallographic directions. For instance, in the case of as-built samples, these strains were oriented along the $\:\left[\stackrel{-}{1}00\right]$ and $\:\left[010\right]$ directions for both types of specimens from scanning and building orientations (see Fig. 11). Whereas in the case of T6 heat-treated samples, the strain components were oriented along the $\:\left[1\stackrel{-}{1}\stackrel{-}{1}\right]$ and $\:\left[010\right]$ directions for specimens in scanning orientation, and the $\:\left[\stackrel{-}{1}00\right]$ and $\:\left[010\right]$ directions for specimens in the building orientation (see Fig. 12). The visualization of the observed strain fields was enhanced through a color-coded scale, which distinctly marked areas of tensile and compressive strains, aiding in a clearer understanding of the material’s deformation behavior. The as-built samples exhibited a combination of tensile and compressive strains at the boundaries in specific crystal directions. In contrast, the T6 heat-treated samples predominantly showed tensile strains in the building orientation and compressive strains in the scanning orientation. Moreover, the range of residual strains in the T6 heat-treated samples was noticeably narrower than in the as-built samples. The error in the presented experimental strains using the 4DSTEM technique has been estimated to be roughly 3.5%, and the error estimation method has been reported in another study^45,46^.

The observed difference in the strain fields of as-built and T6 heat-treated samples can be attributed to the diffusion of alloying elements from Si eutectic regions into Al matrix regions and then the eventual formation of the precipitates in the matrix. For the as-built samples, tensile and compressive strains are concentrated in the eutectic Si boundaries; for the case of T6 heat-treated samples, the higher value strain fields in the vicinity of precipitates were observed, which are likely due to the coherent nature of the precipitates. It is known that the strain coherency at the precipitate/matrix interface induces strains that significantly influence the movement of dislocations within the alloy, thereby impacting its overall mechanical properties.

**Fig. 13:**
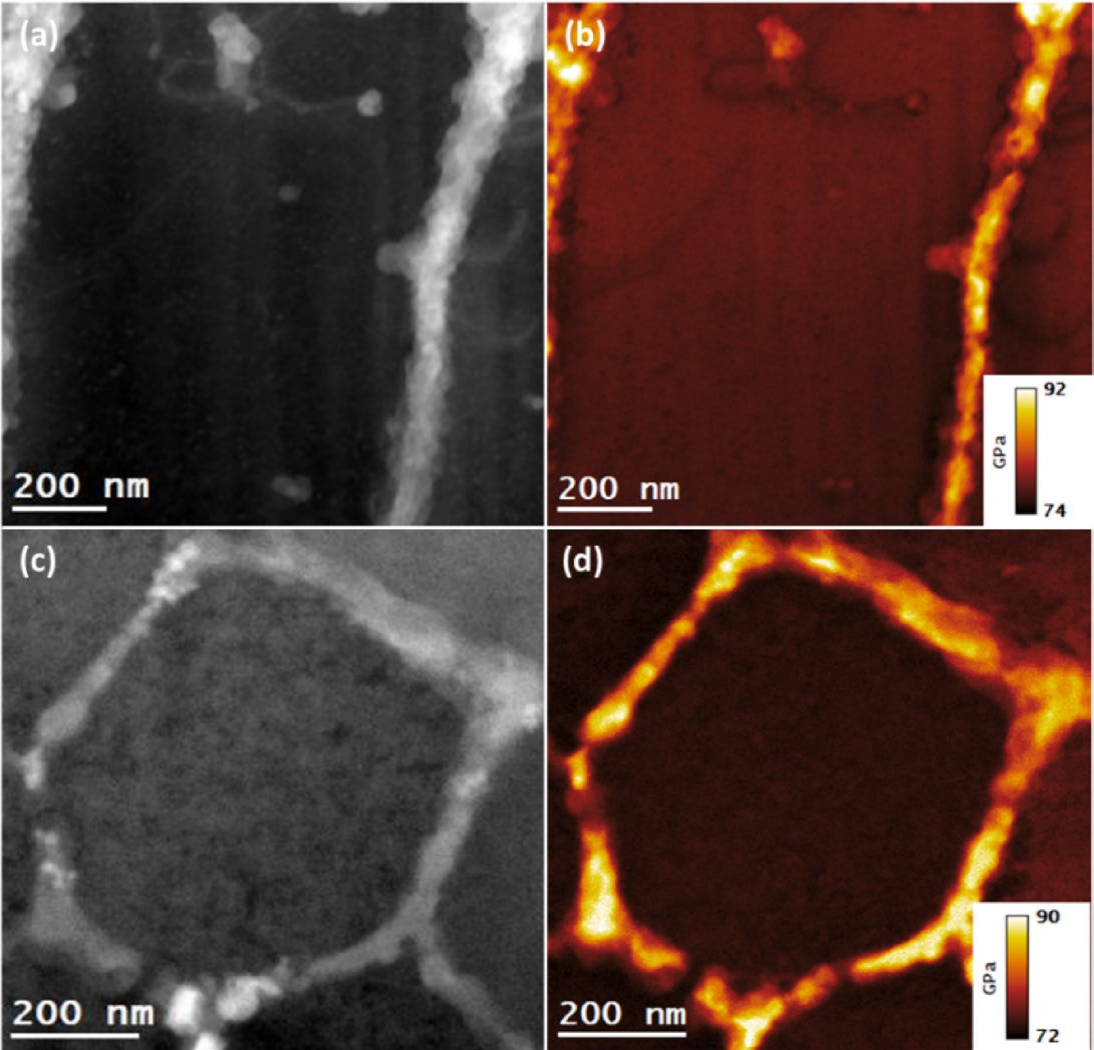
STEM-EELS mapping of Young’s modulus ($\:{Y}_{M}$) in as-built AlSi10Mg alloy samples from specimens in building and scanning orientations. (**a**), and (**c**): DF-STEM images showcasing the regions selected for $\:{Y}_{M}$ mapping. (**b**), and (**d**): $\:{Y}_{M}$ maps along $\:{\left[001\right]}_{Al}$ direction of specimens from building and scanning orientations.

**Fig. 14:**
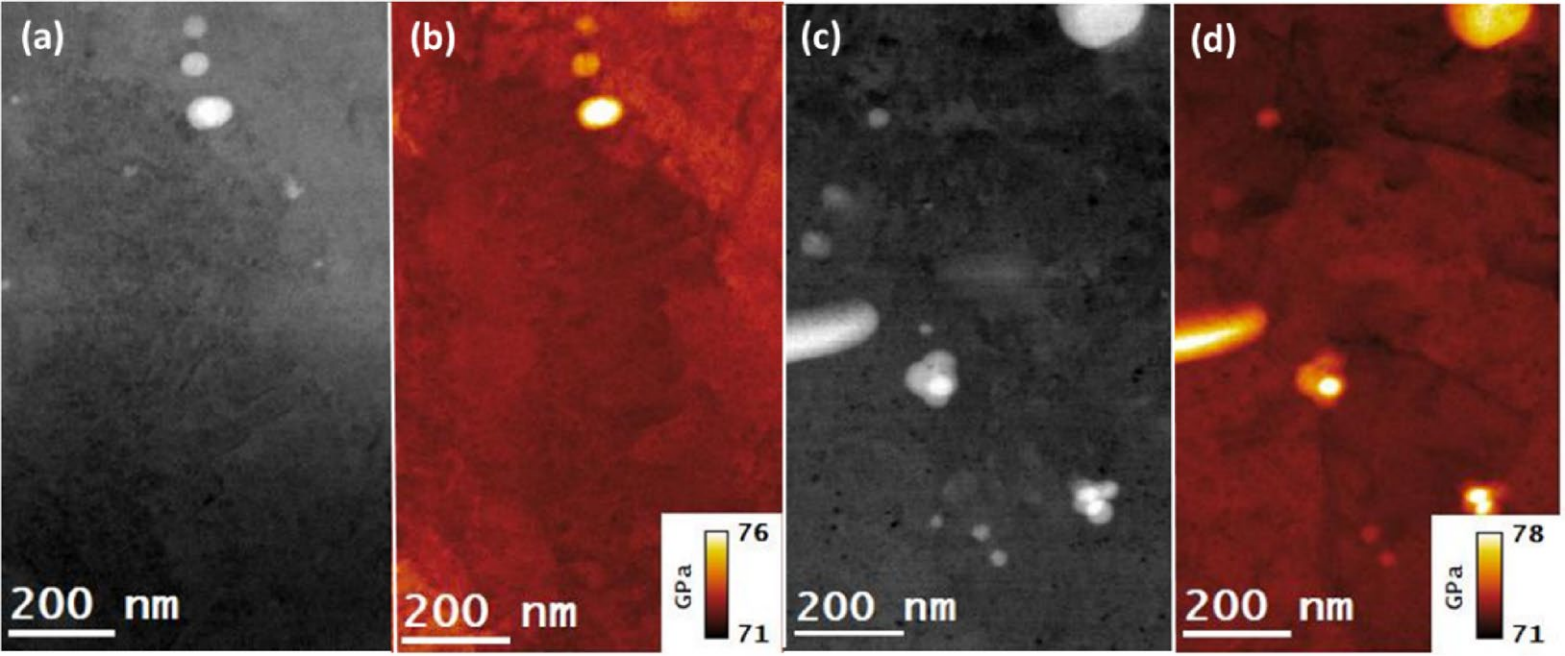
STEM-EELS mapping of Young’s modulus ($\:{Y}_{M}$) in T6 heat-treated AlSi10Mg alloy samples from specimens in building and scanning orientations. (**a**), and (**c**): DF-STEM images showcasing the regions selected for $\:{Y}_{M}$ mapping. (**b**), and (**d**): $\:{Y}_{M}$ maps along $\:{\left[001\right]}_{Al}$ the direction of specimen building and scanning orientations.

The STEM-EELS analysis of as-built, and T6 heat-treated samples was carried out to generate Young’s modulus ($\:{Y}_{M}$) maps from the same regions were analyzed for the determination of residual strain, elemental distributions, and microstructure of the alloys. The analysis was carried out on both specimens from building and scanning orientations, and in each case, the specimens were tilted along the $\:{\left[001\right]}_{Al}\:$zone axis. In this way, a penetrating electron beam generates bulk plasmons acting as longitudinal oscillators at a specific energy ($\:{E}_{p}$), also along the $\:{\left[001\right]}_{Al}$ direction. In this way, the energy of bulk plasmons ($\:{E}_{p}$), can be determined by recording the EELS spectra in the low-loss range while acquiring STEM-EELS datasets. The performed STEM-EELS analysis enabled determining the *E*_*p*_ values of bulk plasmons at each image-pixel and was then utilized to calculate the corresponding value of the $\:{Y}_{M}$ under the Eq. (3). The applied analysis showed an average *E*_*p*_ of about 14.9 eV in the Al matrix area, which was consistent with the existing literature value of the bulk plasmons for the Al metal^46^,. At the same time, it was found to be approximately 15.5 eV in the eutectic Si region. As mentioned earlier, the $\:{Y}_{M}$ maps were generated as per Eq. (5), and the results are presented in Figs. 13 and 14 for both as-built and T6 heat-treated samples, respectively. The uncertainty associated with the experimentally determined $\:{Y}_{M}$ using the STEM-EELS technique is estimated to be approximately 0.5%. The methodology employed for this error estimation has been detailed in a separate study^45,46^. Moreover, the $\:{Y}_{M}$ maps were generated in specimens from building and scanning orientations for each sample. As the specimens were tilted along $\:{\left[001\right]}_{Al}\:$direction, the generated maps also reflect the projection of $\:{Y}_{M}$ along $\:{\left[001\right]}_{Al}$ direction.

The range of color bars in the images is presented in Fig. 13 (b) and (d) showcases $\:{Y}_{M}$ spanning from 74 to 92 GPa for as-built samples in the building orientation and 72–90 GPa in the scanning orientation. Similarly, in the case of T6 heat-treated samples, the range of $\:{Y}_{M}$ was found to be from 71 to 76 GPa in the building orientation and 71–78 GPa in the scanning orientation (see Fig. 14 (b) and (d)). It can be noted from the presented results that the range of $\:{Y}_{M}$ is narrower for heat-treated samples compared to their as-built counterparts. Moreover, the results show a significant difference in $\:{Y}_{M}$ at the Si eutectic regions for as-built samples and Si precipitates in T6 heat-treated samples compared to the matrix regions. It implies that the interatomic bonding among Al-Al atoms becomes stronger at the interface of boundary-matrix and precipitate-matrix due to strain, composition, and dislocation factors.

It is worth noting that the DF-STEM images in Figs. 9 , 11, and 13 represent the same regions of as-built samples from where the acquisitions of STEM-EDS, 4DSTEM, and STEM-EELS datasets were carried out. In this way, for as-built samples, the elemental compositions, residual strains, and Young’s moduli were mapped from the same regions of both scanning and building orientation specimens. Similarly, for T6 heat-treated samples, the DF-STEM images in Figs. 10,12, 14 demonstrate that their elemental compositions, residual strains, and Young’s moduli were also mapped from the same regions in building and scanning orientations. The mapping of $\:{Y}_{M}$ and residual strain mapping from the same areas is critical for determining the residual stresses in the alloy at the nanoscale. This is why great care must be taken to match the coordinates of the strain map with the coordinates of the corresponding $\:{Y}_{M}$ map. In this way, the effect of the dynamic nature of $\:{Y}_{M}$ at the nanoscale can be determined on residual stress maps derived from the corresponding strain maps.

**Fig. 15:**
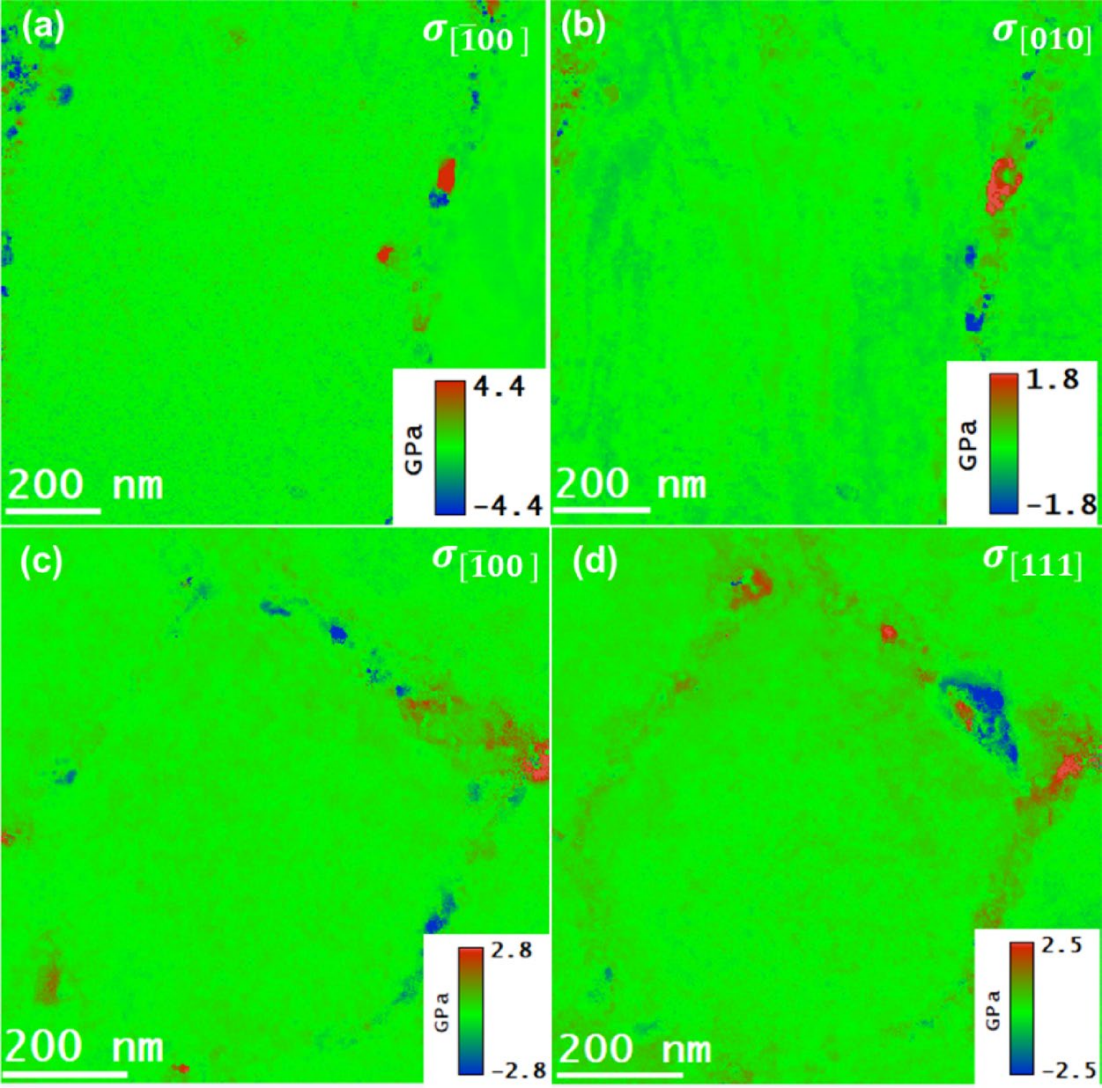
The residual stress maps of as-built AlSi10Mg samples acquired by multiplying the $\:{Y}_{M}$ maps and the corresponding residual strain maps. (**a**), (**b**): normal stresses in the [$\:\stackrel{-}{1}$00] and $\:\left[010\right]$ directions in the building orientation. (**c**), (**d**): normal stresses in the [$\:\stackrel{-}{1}$00] and $\:\left[010\right]$ in scanning orientation.

**Fig. 16:**
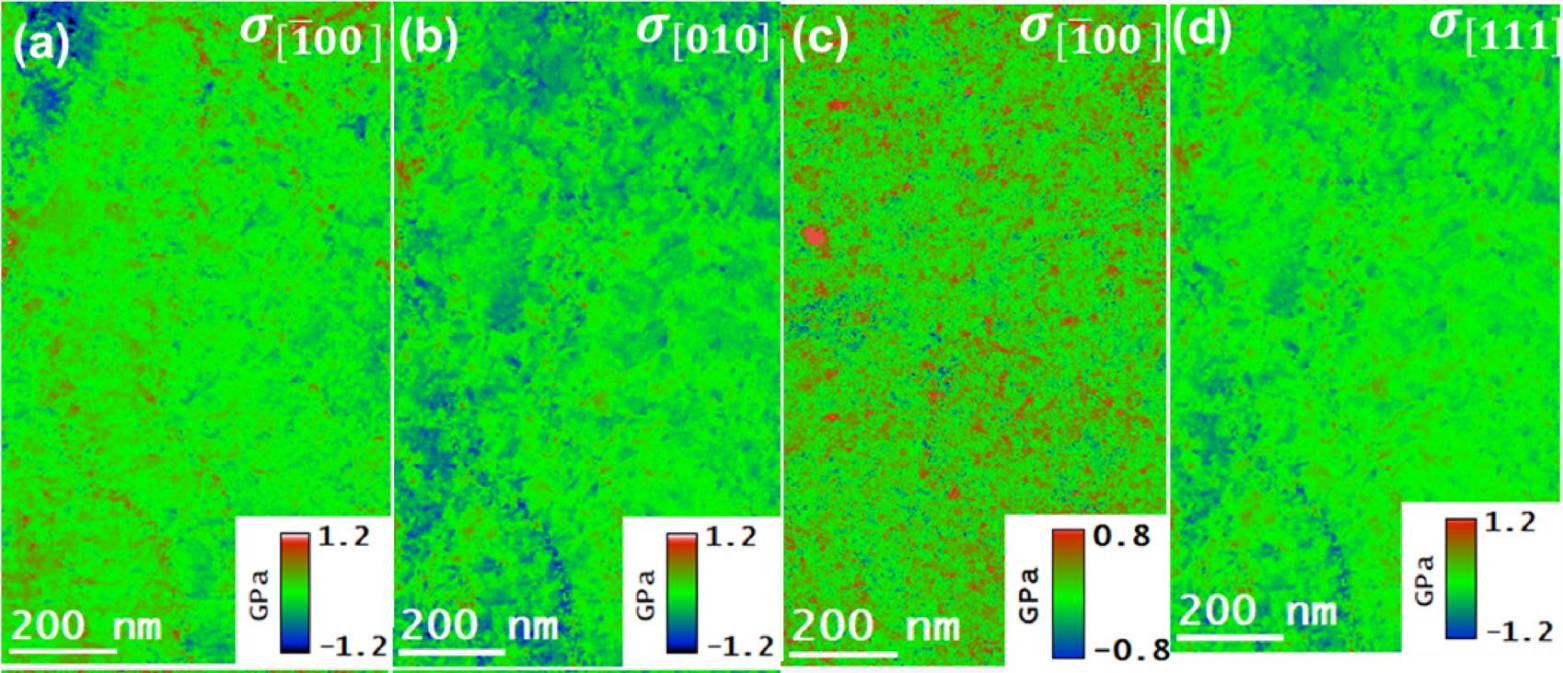
The residual stress maps of T6 heat-treated AlSi10Mg samples acquired by multiplying the $\:{Y}_{M}$ maps and the corresponding residual strain maps. (**a**), (**b**): normal stresses in the [$\:\stackrel{-}{1}$00] and [1$\:\stackrel{-}{1}1$] directions in the building orientation. (**c**), (**d**): normal stresses in the [$\:\stackrel{-}{1}$00] and $\:\left[010\right]$ in scanning orientation.

The residual stress maps of as-built samples were obtained by combining the strain maps presented in Fig. 11 with the corresponding cap Y sub cap M map in Fig. 13, under Hooke’s law, as represented in Eq. (4). Similarly, the residual stress maps for T6 heat-treated samples can be determined by combining the strain map in Fig. 12 with Young’s modulus map in Fig. 14. The results obtained on the residual stress maps for as-built and T6 heat-treated samples are presented in Fig. 15, and 16, respectively. Moreover, the presented results on both samples contained stress mapping performed on specimens from building and scanning orientations. It can be noticed from the generated stress maps that the range of residual stresses in the as-built samples was more extensive than that of the T6 heat-treated samples. For instance, in the tensile and compressive regions, the average residual stresses (<$\:{\sigma\:}_{ii}$>) in $\:\left[\:\stackrel{-}{1}00\right],$ $\:\left[010\right]$, and $\:\left[11\stackrel{-}{1}\right]$ directions are addressed next. For the as-built samples, the tensile and compressive stresses are concentrated in the eutectic Si boundaries; for the case of T6 heat-treated samples, the higher value strain fields in the vicinity of residues were observed, which are likely due to the coherent nature of the precipitates. Table 2 summarizes the average residual stress values in both tensile and compressive regions for different crystallographic directions, including the $\:\left[\:\stackrel{-}{1}00\right],$ $\:\left[010\right]$, and $\:\left[11\stackrel{-}{1}\right]$ directions, the latter being relevant for the T6 heat-treated samples in the scanning orientation.

The qualitative anisotropy in residual stresses in the samples was quantitatively determined by determining the average stresses in a specific crystallographic direction. For as-built samples, the average residual stress in the tensile regions along the $\:\left[\stackrel{-}{\:1}00\right]$ direction decreased from 2.05 GPa in the building orientation to 1.78 GPa in the scanning orientation. Marola et al. reported residual stress value for the as-built sample quantified using mana spectroscopy, higher than 1 GPa^47^. In contrast, the T6 heat-treated samples reduced from 0.56 GPa in the bundling orientation to 0.43 GPa in the scanning orientation.

**Table 2:** Average residual stress ($\:\langle{\sigma\:}_{ii}\rangle$) values obtained from the tensile and compression regions.

Specimens	RS in tensile regions (GPa)		RS in compressive regions (GPa)	
	$\:\langle{\sigma\:}_{\left[\stackrel{-}{1}00\right]}\rangle$	$\:\langle{\sigma\:}_{\left[010\right]}\rangle$	$\:\langle{\sigma\:}_{\left[\stackrel{-}{1}00\right]}\rangle\:$	$\:\langle{\sigma\:}_{\left[010\right]}\rangle$
From as-built sample in building orientation	2.05	1.69	−2.23	−1.19
From as-built sample in scanning orientation	1.78	1.65	−1.92	−1.94
From T6 heat-treated sample in building orientation	0.56	0.58	−0.64	−0.71
From T6 heat-treated sample in scanning orientation	0.43	0.39	−0.39	−0.62

In the compressive regions, the average residual stress increased from − 2.23 GPa to −1.92 GPa in the scanning orientation for as-built samples and from − 0.64 GPa in scanning orientation to −0.39 GPa in the building orientation for T6 heat-treated sample. Such a variation in residual stress values across different orientations suggests the presence of anisotropy at the nanoscale. This anisotropy mirrors the behavior observed at larger scales, confirming the consistency of these stress patterns across different scales^48^. The residual stress maps are in the range of the reported residual stress values in the literature^47,49,50^. Zhang et al.^46^ provide strong evidence that nano-sized Si particles formed in AlSi10Mg via PBF-LB processing significantly contribute to Orowan-type strengthening, with in-situ mechanical testing revealing peak stress values approaching ~ 2 GPa. This finding aligns well with our observations. In our study, nanoscale stress mapping using 4DSTEM and STEM-EELS techniques revealed residual stresses of similar magnitude (~ 2 GPa, in both tension and compression) localized within Si-rich regions in both as-built and T6 heat-treated samples. These elevated stress concentrations support the notion that nano-sized Si precipitates not only reinforce the matrix through Orowan strengthening but also serve as localized stress concentrators. This dual role likely enhances their impact on the residual stress distribution and mechanical behavior, particularly through dislocation pinning effects, which are comparable to those observed during active deformation.

The alloys’ residual stresses decreased significantly due to the T6 heat treatment, ranging from 13 to 23%, depending on the sample orientation. This decrease is accompanied by a reduction in the alloy’s anisotropy. These observations indicate that the alloy undergoes defect annealing, which ultimately reduces the density of the crystal defects, such as point and line defects.

**Fig. 17:**
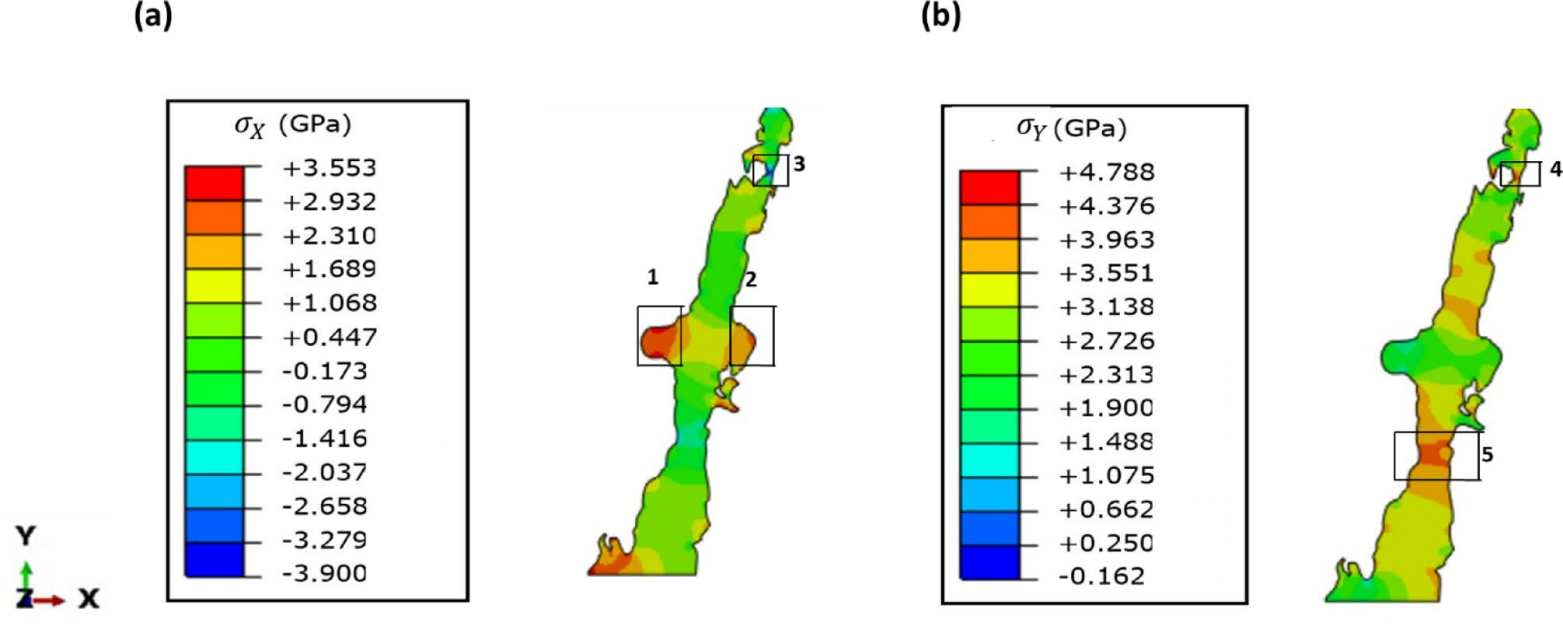
Residual stress maps obtained from the FEM model. (**a**) Stresses in X direction ($\:{\sigma\:}_{X}$), and (**b**) Stresses in Y direction ($\:{\sigma\:}_{Y}$).

As mentioned earlier, the image-based FEM was developed to simulate the residual stresses occurring in the as-built AlSi10Mg alloy samples due to their heat treatment. Figure 17 presents the simulated residual stress maps in Si eutectic regions in X or [$\:\stackrel{-}{1}$00] and Y or [010] directions. For both directions, the model revealed the presence of significant stress gradients in the entire Si eutectic region. In particular, the gradients were much higher in some distinct areas, such as 1, 2 in the X-direction and 4 and 5 in the Y-direction. Quantitatively, the tensile stress values at 1, 2, 4, and 5 regions were measured to be around 3.2 GPa, 2.5 GPa, 4.1 GPa, and 4.6 GPa, respectively. Similarly, region 3 contained pronounced compressive stresses of −3.3 GPa. These stress values indicate a potential fracture along the upper boundary, separating it from the lower part. Such stress-induced diffusion is compounded with temperature-controlled diffusion.

The stress and concentration-driven diffusion mechanisms will result in spheroidization and, ultimately, breaking Si eutectic regions or zones. The overall diffusion process leads to uniformity in Si concentration and stress fields. For instance, the elemental maps in Fig. 10 shows a dramatic dissolution of Si at the eutectic zone in T6 heat-treated samples compared to as-built samples. However, the dissolution of Fe at the eutectic regions is not that dramatic, which implies that the effect on stress distribution in the matrix is higher for Si than in the case of Fe. In the same way, Fig. 17, also shows a dramatic decrease in the stress gradients at the eutectic regions. This model is a significant step forward in understanding the complex stress patterns that may occur during the heat treatment of as-built AlSi10Mg alloys, which lead to the break of the Si eutectic.

It is worth mentioning that the role of elemental composition of solute elements in metal alloys is quite important for addressing the variations observed in $\:{Y}_{M}$, strains, and residual stress maps. Figures 9 and 10, present the elemental composition of both the as-built and heat-treated samples, respectively. Specifically, it was observed that the $\:{Y}_{M}$ is higher around Si eutectic and precipitates regions in comparison to the aluminum (Al) matrix. This is because of the presence of Si, Mg, and Fe atoms. This implies that Al-Al bonds are stronger near the silicon eutectic and precipitates, possibly mediated by Al bonding with other elements. In other words, this finding also helps the presence of coherent boundaries between the Al matrix and Si eutectic regions.

The methodologies for determining residual stresses within a single grain at nanoscale resolution in AM AlSi10Mg alloys cannot be commonly found in the literature. Nonetheless, using Raman spectroscopy, Morla et al.^47^, measured the residual stress in a PBF-LB fabricated AlSi10Mg alloy. Stress levels as high as in the range of GPa were reported in the presented study, which was also reported in the literature^47,49,50^. Furthermore, these findings enabled determining the presence of anisotropy in the residual stresses. Therefore, the method introduced in this study presents an effective way of mapping and quantifying residual stress and detecting anisotropy in additively manufactured metal alloys within a single grain. In this way, the results obtained with the presented methodology will enable quantification and optimization of residual stress for the processes utilized when manufacturing metal alloys for industrial applications.

The generation of residual stress maps for AlSi10Mg alloys involves combining strain maps obtained via 4D-STEM with modulus data determined through STEM-EELS ($\:{Y}_{M}$). This approach provides insights into how residual stress influences the fatigue behavior and damage tolerance of these alloys, which are critical for industrial applications. A qualitative analysis of the impact of this methodology is presented herein. It is carried out by generating the residual stress maps obtained by multiplying a constant $\:{Y}_{M}$ value with a strain map, then $\:{Y}_{M}$ map with the same strain map. It was found that there is about a 2% mismatch between the two cases, and it highlights the fact that adopting the$\:{Y}_{M}$ map approach improves the determination of residual stresses. This will surely improve the fatigue and damage tolerance levels for the alloys, which determine the reliability of alloys mainly used in the aerospace industry. It is known that the aerospace industry requires alloys to be highly reliable^51^. Therefore, the residual stress mapping obtained by combining the strain maps with $\:{Y}_{M}$ map will improve the reliability of data quality for AlSi10Mg alloys. The change in residual stresses due to the variable $\:{Y}_{M}$ will quite expectedly affect the fatigue life of the alloy. Expressly, the effect on fatigue life can be understood through the stress-cycle (SN) curves on the alloys^52,53^. Therefore, depending upon an increase or decrease in residual stress, the number of cycles as a function of applied stress will change, at which fatigue will occur in the alloy. In other words, the presented $\:{Y}_{M}\:$map-based methodology for determining residual stress may impact industries like the aerospace industry, where the residual stresses must be determined with high precision for accurate fatigue analysis.

## **Conclusions**

In mutually perpendicular laser scanning and alloy building orientations, correlated STEM-based SI techniques of TEM provide a novel way to map residual stresses within a single grain at nanoscale spatial resolution in PBF-LB-fabricated AlSi10Mg alloy. To map residual strains and Young’s modulus from the same regions of the alloy samples, 4DSTEM and STEM-EELS techniques can be used. At the same time, the STEM-EDS method allows mapping the elemental distributions in the alloy samples, which are crucial to understanding the thermally induced processes that eventually control the alloy’s precipitate formation. The resulting residual stress maps can be further post-processed to investigate the anisotropy of PBF-LB-fabricated alloys. For example, the proposed scheme showed that the stress anisotropy between the laser scanning and building orientations was about 23%. Still, it decreased by 13% when the as-built samples were subjected to T6 heat treatment. Including Young’s modulus maps as supposed to be a constant value increases the accuracy of the alloys’ residual stress maps, which may be necessary because a better prediction about alloys’ fatigue life can be made in this way. The image-based FEM simulations proved to be an effective tool for estimating extreme residual stress regions in AlSi10Mg alloy, particularly within the Si eutectic zones. Such simulations help to identify the extreme stress regions that are a potential source of Si diffusion into the alloy matrix. In conclusion, anisotropy in PBF-synthesized AlSi10Mg alloys underscores the critical need to determine anisotropy using any technique, including electron microscopy-based techniques. Future work will include a detailed dislocation analysis to further understand the local plasticity behavior associated with residual stress evolution in PBF-LB processed AlSi10Mg. Additionally, the method presented for AlSi10Mg characterization will potentially refine the processes effectively to produce PBF-synthesized alloys in the metal industry.

## Data Availability

All the data generated or analyzed throughout this study are provided within the published article and can be provided upon request. Contact the Corresponding author (Dr. Dalaver Anjum, dalaver.anjum@ku.ac.ae).
